# Supplementary material for: Can creative activities and mind-body practices help enhance well-being and mental health awareness? An exploratory qualitative study in UK higher education
Source: PLoS One. 2025 Dec 17;20(12):e0338938. doi: 10.1371/journal.pone.0338938 (PMC12711057; doi:10.1371/journal.pone.0338938)
Supplement: Attachment — Submitted filename: Response to reviewers.docx [file pone.0338938.s001.docx]

Rebuttal letter

The following summarizes the revisions made in response to the reviewer’s comments and the journal’s guidance.

*Journal comments*

1) Comment: Please ensure that your manuscript meets PLOS ONE's style requirements, including those for file naming.

Response: The manuscript was reviewed against the journal’s guidelines, and minor adjustments were made, particularly to paragraph indentation, to ensure full compliance with the guidelines.

2) Comment: Please note that funding information should not appear in any section or other areas of your manuscript. We will only publish funding information present in the Funding Statement section of the online submission form. Please remove any funding-related text from the manuscript.

Response: Any mention of funding has been removed from the manuscript (previously in the Acknowledgements section).

3) Comment: Please state what role the funders took in the study. If the funders had no role, please state: "The funders had no role in study design, data collection and analysis, decision to publish, or preparation of the manuscript."

Response: A ‘Role of funders’ statement has been added to the Cover Letter clarifying that the funders had no role in the research or publication.

4) Comment: Before we proceed with your manuscript, please also provide non-author contact information (phone/email/hyperlink) for a data access committee, ethics committee, or other institutional body to which data requests may be sent. If no institutional body is available to respond to requests for your minimal data, please consider if there any institutional representatives who did not collaborate in the study, and are not listed as authors on the manuscript, who would be able to hold the data and respond to external requests for data access? If so, please provide their contact information (i.e., email address). Please also provide details on how you will ensure persistent or long-term data storage and availability.

Response: The ‘Data availability statement’ has been updated to provide an institutional contact external to the authors - the Ethics Committee that approved the research project. One of the authors currently serves as co-chair of the committee; however, at the time of approval (2023), they had no involvement with the committee. Access to the data is managed by the Ethics Committee, not by the individuals serving as chairs, which ensures independence. The current co-chairs, including the author, are aware that the committee itself, through its institutional processes, holds custody of the data. The co-chair role rotates every three years. Data are securely stored on the University’s cloud storage and shared with the Ethics Committee, ensuring long-term availability.

5) Comment: Please include your full ethics statement in the ‘Methods’ section of your manuscript file. In your statement, please include the full name of the IRB or ethics committee who approved or waived your study, as well as whether or not you obtained informed written or verbal consent. If consent was waived for your study, please include this information in your statement as well.

Response: A comprehensive ethics statement has been added to the ‘Materials and Methods’ section, at the start of the ‘Ethical Procedure’ sub-section.

*Reviewer comments*

1) Comment: Clarify limitations. Explicitly acknowledge the limited sample size and self-selection bias, and explain how these may affect the transferability of findings.

Response: A dedicated section on limitations has been added following the ‘Discussion’. This section explicitly acknowledges self-selection bias, potential inaccuracies inherent in self-reported data, the small sample size, and its implications for the generalizability of the findings.

2) Comment: Refine data availability statement.

Response: The ‘Data availability statement’ has been updated in alignment with the reviewer (and the journal)’s comment.

3) Comment: Streamline sections of the manuscript. Consider condensing the literature review and parts of the discussion where points are repeated, focusing on the most salient findings and implications.

Response: Sections of the ‘Discussion’ have been streamlined, reducing the word count from approximately 1300 to 900 words, and the discussion of findings has been presented in a more integrated manner to enhance readability and impact. The Literature review (Introduction) has not been further shortened (remaining around 500 words), as it was already heavily streamlined during manuscript drafting. The authors believe that additional cuts could compromise the clarity of the research rationale and weaken the demonstration of a thorough review of relevant literature.

4) Comment: Broaden the implications. Consider including a brief reflection on how these findings might inform institutional policy beyond the immediate context (e.g., implications for other universities or community-based programs).

Response: The Conclusions section has been revised to broaden the implications of our findings, highlighting their relevance for other higher education institutions and community-based programs.

5) Comment: Address researcher positionality. While insider status is acknowledged, more discussion of how this was managed during data collection and analysis (e.g., reflexive journaling or audit trails) would strengthen trustworthiness.

Additional details have been added to both the ‘The Insider Researcher’ and ‘Data Analysis’ sections to clarify how we managed our positions as insider researchers and ensured the trustworthiness of the findings.
