# Supplementary material for: Can creative activities and mind-body practices help enhance well-being and mental health awareness? An exploratory qualitative study in UK higher education
Source: PLoS One. 2025 Dec 17;20(12):e0338938. doi: 10.1371/journal.pone.0338938 (PMC12711057; doi:10.1371/journal.pone.0338938)
Supplement: Attachment — Submitted filename: Response_to_reviewers_auresp_2.docx [file pone.0338938.s002.docx]

Rebuttal letter

The following summarizes the revisions made in response to the reviewers’ comments and the journal’s guidance.

*Reviewer 2 comments*

1) Comment: Expand the limitations section to discuss how questionnaire format may limit depth and suggest future mixed-methods follow-ups.

Response: The limitations of the questionnaire format have been addressed in the ‘Limitations’ section, explicitly noting the potential constraints on response depth and recommending that future research adopt a mixed-methods approach

2) Comment: Add a brief subsection in discussion on how findings align with or diverge from US studies cited, to strengthen the UK gap argument.

Response: At the beginning of the Discussion section, a brief summary has been added to highlight how the findings of this study both align with and differ from US-based research. In particular, the shared positive effects of creative activities and mind-body practices are acknowledged, while the distinction between the controlled interventions typical of some US studies and the investigation of pre-existing activities in this UK-based study is clearly noted.

3) Comment: Consider including a supplementary file with the full questionnaire for transparency.

Response: Unfortunately it is not possible to include the full questionnaire as a supplementary file, as it contains identifying references that could, directly or indirectly, compromise the privacy of individuals involved in the activities. However, as added in the Data Availability statement, a copy of the questionnaire can be provided upon reasonable request.

*Reviewer 3 comments*

1) Comment: Under methodology, the study design is not made clear, this needs to be stated clearly.

Response: In the ‘Study Design’ section we added a statement that clarifies why the questionnaire was chosen as the data collection tool as opposed to others such as interviews. The ‘Materials and Methods’ section, along with its subsequent subsections, addresses all key components of the design, including the research aim and rationale, the methodological approach (qualitative phenomenological design), data collection, participant selection, and data analysis. The authors, supported by feedback from the first reviewer, consider the study design to be clear and consistent with reporting practices in recent qualitative publications in PLOS ONE (e.g. [Khan et al., 2025](https://journals.plos.org/plosone/article?id=10.1371/journal.pone.0320088)).

2) Comment: Under results section, last column of Table 2 is not clear; it needs to be made clear.

Response: The last column of Table 2 has been revised for clarity to indicate that participants did not respond to the question addressed in the Table. In the staff responses, ‘0’ has been used instead of ‘N/A’ to avoid confusion.
